# Research Trends and Emerging Themes in Gut Microbiota–Systemic Lupus Erythematosus Research: A Bibliometric Analysis (2014-2025)

**DOI:** 10.5152/ArchRheumatol.2026.25284

**Published:** 2026-06-03

**Authors:** Jun-an Yang, Ji-an Wang

**Affiliations:** 1 Department of Rheumatology and Immunology, Xinchang Hospital of Traditional Chinese Medicine, Shaoxing, China; 2 Department of Rheumatology and Immunology, The First Affiliated Hospital of Zhejiang Chinese Medical University (Zhejiang Provincial Hospital of Chinese Medicine), Hangzhou, China

**Keywords:** Bibliometric analysis, gastrointestinal microbiome, Scopus, systemic lupus erythematosus, web of science

## Abstract

**Background/Aims:**

Growing evidence indicates that gut microbiota dysregulation plays a crucial role in the pathogenesis of systemic lupus erythematosus (SLE); yet, a quantitative overview of research activity in this field is lacking. This study aimed to provide a bibliometric analysis of global research trends and thematic evolution concerning gut microbiota and SLE between 2014 and 2025.

**Materials and Methods:**

Relevant publications were retrieved from the Web of Science Core Collection and Scopus databases, and only original research articles and review articles published in English between January 1, 2014, and December 31, 2025, were included. Bibliometric analyses were performed using the bibliometrix package in R and CiteSpace to evaluate publication trends, country and institutional contributions, collaboration networks, keyword co-occurrence, and citation burst patterns.

**Results:**

A total of 525 publications were included, comprising 248 original articles and 277 reviews. Annual publication output increased steadily, with accelerated growth after 2019 (annual growth rate of 121.20%), and 91 papers were published in 2025. China and the United States were the main contributors, accounting for more than half of the total publications, with field-normalized contributions of 1.0927 and 1.2268. The Medical University of South Carolina and Zhejiang Chinese Medical University were the most productive institutions (10 articles). Citation burst analysis showed that “Intestinal dysbiosis associated with systemic lupus erythematosus” laid the foundation for research in this field. Keyword analysis indicated a gradual shift from descriptive microbial characterization toward themes related to immune regulation and dysbiosis. In addition, increasing research attention has been directed toward microbiota-based therapeutic approaches in SLE.

**Conclusion:**

Research on gut microbiota in SLE has expanded substantially over the past decade, with an observable shift from association-based studies toward more mechanistic and translational research themes. Distinct regional research emphases were noted, highlighting the potential value of enhanced international and interdisciplinary collaboration in advancing research in this field.

**Main Points**

- Global research on gut microbiota and systemic lupus erythematosus has increased markedly since 2019.
- Research focus has shifted from microbial profiling toward immune regulation and dysbiosis-related mechanisms.
- Distinct national research patterns emphasize the importance of international collaboration.

## Introduction

Systemic lupus erythematosus (SLE) is a chronic, multisystem autoimmune disease characterized by dysregulated immune activation, autoantibody production, and widespread inflammatory organ damage.^^1^^^^-^^^^3^^ The epidemiology of SLE exhibits pronounced geographic and sex-specific disparities, with a global prevalence estimated at 40-200 per 100 000 individuals and a markedly higher incidence among women, particularly those of reproductive age, highlighting the contributory role of hormonal factors in disease susceptibility.^^4^^^,^^^5^^ Clinically, SLE is highly heterogeneous, presenting with manifestations ranging from cutaneous involvement and arthritis to lupus nephritis and neuropsychiatric complications.^^6^^^,^^7^ Its pathogenesis is multifactorial, involving genetic predisposition (e.g., HLA-DR, IRF5, STAT4), epigenetic dysregulation, and environmental triggers such as ultraviolet exposure, viral infections, and chemical agents.^^8^^, ^-^, ^10^,^^11^^

The gut microbiota represents the largest and most metabolically active microbial ecosystem in the human body and plays a fundamental role in immune homeostasis.^^12^^ Through the production of microbial metabolites, modulation of immune cell differentiation, and maintenance of intestinal barrier integrity, gut microbiota critically shapes host immune responses.^^13^^^,^^^14^^While a substantial proportion of the gut microbial community remains relatively stable over time, microbial composition can be dynamically influenced by age, diet, lifestyle, and environmental exposures.^^15^^^,^^^16^^Perturbations in this ecosystem have been increasingly implicated in the development and progression of immune-mediated and metabolic disorders, including SLE.^17^^-^^20^

In recent years, a growing body of experimental and clinical evidence has highlighted a close association between gut microbiota dysbiosis and SLE pathogenesis. Preclinical studies have demonstrated that alterations in microbial composition can promote autoantibody production, with reductions in Lachnospiraceae and enrichment of Enterobacteriaceae correlating positively with disease activity indices such as SLEDAI.^^21^^^,^^^22^^ Metagenome-wide association studies further revealed disease-specific microbial signatures in patients with SLE, including increased abundance of *Streptococcus anginosus* and *Streptococcus intermedius*, accompanied by functional shifts in pathways related to sulfur metabolism and flagellar assembly.^^23^^ Moreover, germ-free and fecal microbiota transplantation models have provided compelling causal evidence, showing that transplantation of SLE patient-derived microbiota can induce autoimmune phenotypes in recipient mice, whereas probiotic interventions—particularly *Lactobacillus* species—can ameliorate lupus nephritis.^^24^^^,^^^25^^

Although a previous scientometric study has examined microbial dysbiosis in SLE,^^26^^ dysbiosis represents an important dimension of gut microbiota research. A comprehensive and updated bibliometric evaluation encompassing the broader SLE–gut microbiota research landscape, integrating multiple databases and expanded analytical indicators, remains warranted. Bibliometric analysis, which applies quantitative and statistical methods to evaluate patterns in scientific publications, offers an objective framework to characterize research productivity, collaboration networks, knowledge structures, and emerging trends.^^27^^ The absence of such analyses in the SLE–gut microbiota domain hampers a comprehensive understanding of its developmental trajectory and limits evidence-based planning for future research directions.

Therefore, the present study conducted a bibliometric analysis of publications indexed in the Web of Science Core Collection from 2014 to 2025 to systematically map the global research landscape in this field. Specifically, the study aimed to address the following research questions: (1) How has the annual publication output evolved over time? (2) Which countries, institutions, authors, and journals have made the most influential contributions? (3) What collaboration patterns can be identified at the national and institutional levels? (4) How has the intellectual structure of the field evolved based on co-citation and keyword analyses? (5) What emerging research hotspots and future directions can be identified?

## Materials and Methods

### Data Source and Search Strategy

All publications included in this study were retrieved from the Web of Science Core Collection and Scopus databases. In Web of Science, a search was performed using the following strategy: (“systemic lupus erythematosus” OR “SLE”) (Topic) AND (“gut microbiota” OR “gut microbiome” OR “gut flora” OR “gastrointestinal microbiota” OR “gastrointestinal microbiome” OR “gastrointestinal flora”) (Topic). In Scopus, a TITLE-ABS-KEY search was performed using the following strategy: (TITLE-ABS-KEY (“systemic lupus erythematosus” OR “SLE”) AND TITLE-ABS-KEY (“gut microbiota” OR “gut microbiome” OR “gut flora” OR “gastrointestinal microbiota” OR “gastrointestinal microbiome” OR “gastrointestinal flora”)). The literature search was conducted on February 24, 2026. Original research articles and review articles published in English between January 1, 2014, and December 31, 2025 were included. Only 2 relevant publications were identified in each of the years 2011, 2012, and 2013. Given the extremely low and stable annual output during this period, these early years were considered insufficient to support meaningful trend analysis. Therefore, 2014 was selected as the starting point to ensure greater statistical robustness and reliability in the bibliometric evaluation.

### Data Collection and Preprocessing

Records meeting the inclusion criteria were downloaded from the Web of Science (https://​www.webo​fscience​.com/wos​/alldb/b​asic-sea​rch) and Scopus (https://​www.scop​us.com/p​ages/hom​e#basic) databases with full bibliographic information and cited references. To ensure data accuracy and consistency, all retrieved records were independently screened by 3 researchers according to predefined inclusion and exclusion criteria. Any discrepancies in study selection were resolved through discussion until consensus was reached. Records were excluded if they met the following criteria: (1) conference papers or editorials; (2) content unrelated to SLE and gut microbiota; and (3) duplicate, republished, or retracted literature. All data were exported in plain text format and subsequently processed using the bibliometrix package (version 5.2.1, https://​www.bibl​iometrix​.org/hom​e/) in R software (version 4.5.1, https://​www.r-pr​oject.or​g/) (R Foundation for Statistical Computing; Vienna, Austria) for format conversion and duplicate removal prior to analysis. To reduce inconsistencies in author and institutional names across databases, name harmonization procedures were performed using the bibliometrix package, including normalization of name variants. Major contributing institutions were manually reviewed to ensure consistent attribution and to minimize duplication caused by formatting differences.

### Bibliometric Analysis

Bibliometric analyses were performed to evaluate publication output, citation patterns, and scholarly contributions across countries, institutions, journals, and authors. To provide a relative assessment of national research intensity, field-normalized contribution was calculated by dividing the number of SLE–gut microbiota publications by the total publication output of each country during the same period. This normalization allows comparison independent of overall national research volume. Key indicators, including annual publication counts, H-index, and citation frequency, were calculated using R and the bibliometrix package. The annual growth rate was derived using the bibliometrix package based on changes in publication output over time. Scientific knowledge mapping analyses, including co-citation networks, keyword co-occurrence, and thematic evolution, were conducted using CiteSpace software (version 6.4.R1, https://​citespac​e.podia.​com/) (Massimo Aria; Naples, Italy). Citation burst detection was applied to identify emerging research fronts and evolving hotspots within the field. All statistical analyses and visualizations were generated using R software (R Foundation for Statistical Computing; Vienna, Austria). For network construction, institutions, authors, and keywords with an occurrence frequency greater than 5 were included to improve visualization clarity and reduce noise.

### Ethical Considerations

As this study is a bibliometric analysis based on publicly available data from scientific databases (e.g., Web of Science, Scopus), it did not involve direct interaction with human participants, animal subjects, or access to private clinical data. Therefore, ethical approval from an institutional review board or ethics committee was not required for this type of research. Similarly, informed consent was not applicable as no individual-level data were collected or used.

## Results

### Overview of Publication Output

**Figure 1.**
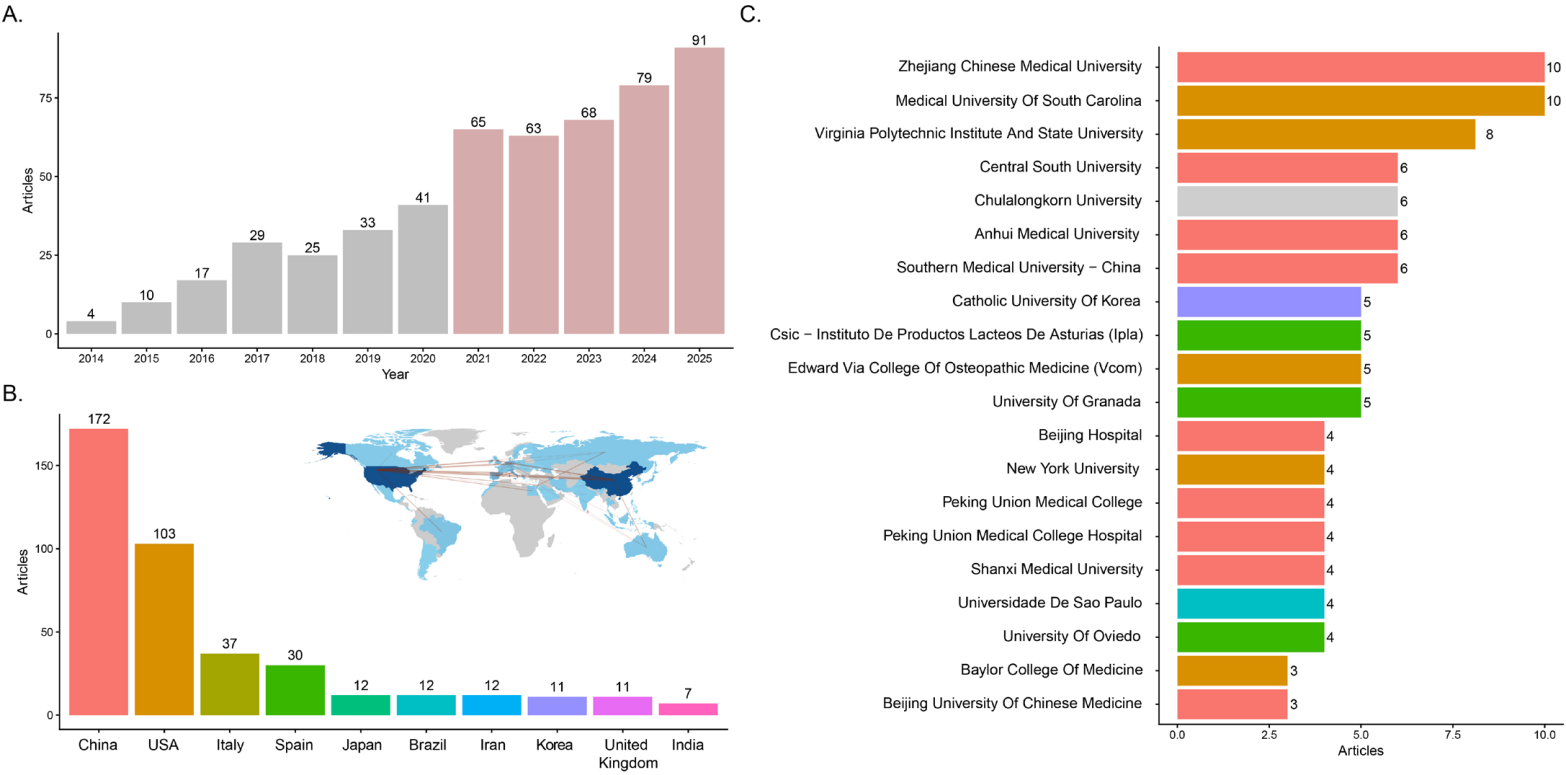
Global publication landscape of research on the association between systemic lupus erythematosus and gut microbiota. (A) Annual publication trends from 2014 to 2025. (B) Geographic distribution of publications by country. (C) Leading institutions contributing to systemic lupus erythematosus–gut microbiota research.

A total of 371 and 431 records were initially retrieved from the Web of Science Core Collection and Scopus databases. After merging records from the 2 databases and excluding records with incomplete information, 525 publications were included for subsequent analysis, comprising 248 original research articles and 277 review articles. As shown in Figure 1A, annual publication output remained below 30 articles from 2014 to 2018, followed by a sharp increase between 2019 and 2025, with an average annual growth rate of 121.20%. After 2021, the number of published papers each year exceeded 60, with the highest number of 91 papers published in 2025. Overall, these findings indicate a rapidly expanding research interest in the association between SLE and the gut microbiota.

### National Contributions and International Collaboration

Analysis of national publication output (Figure 1B) showed that China was the leading contributor with 172 publications, followed by the United States (n = 103), Italy (n = 37), Spain (n = 30), and Japan (n = 12). After normalization to overall national publication volume, China and the United States remained leading contributors. The field-normalized contributions were 1.0927 for China and 1.2268 for the USA, while other countries were all less than 1. International collaboration analysis revealed that China–United States cooperation was the most frequent (21 collaborations), followed by collaborations between the United States and Germany (9), between China and the United Kingdom (5), and between Italy and Spain (5) (Table 1). These results highlight the dominant roles of China and the United States in shaping the global research landscape of this field.

### Institutional Contributions

A total of 370 institutions worldwide contributed to publications related to SLE and gut microbiota. The top 20 most productive institutions are presented in Figure 1C and Supplementary Table 1. The Medical University of South Carolina (USA) and Zhejiang Chinese Medical University (China) were the most productive institutions, each contributing 10 publications, followed by Virginia Polytechnic Institute and State University (USA) with 8 publications. Among the top 20 institutions, 9 were from China and 5 from the United States, indicating strong institutional engagement from both countries.

### Journals and Publication Impact

The included publications were distributed across 258 academic journals, and 19 journals had published more than 5 articles each. The top 10 most productive journals are summarized in Table 2. *Frontiers in Immunology* published the largest number of articles (n = 67), followed by *International Journal of Molecular Sciences* (n = 19) and *Journal of Autoimmunity* (n = 16). Based on the latest available 2024-2025 Journal Citation Reports data, the top 10 journals were all ranked in JCR Q1. Notably, articles published in *Gut Microbes*, *Autoimmunity Reviews*, and *Journal of Autoimmunity* generally exhibited higher citation impact (IF was 11.00, 8.3, and 7.0), suggesting greater academic influence within this research domain.

### Most Cited Publications and Citation Burst Analysis

Citation analysis was performed on 26 017 referenced articles, and the top 10 most cited publications are listed in Table 3. Among them, 3 landmark studies were particularly influential. “The Bidirectional Relationship of Depression and Inflammation: Double Trouble” was the most cited (1,598 citations), providing a theoretical framework linking gut microbiota to neuroinflammation and immune dysregulation relevant to SLE comorbidities. “Does the Epithelial Barrier Hypothesis Explain the Increase in Allergy, Autoimmunity, and Other Chronic Conditions?” (851 citations) proposed a novel hypothesis emphasizing epithelial barrier dysfunction in autoimmune disease pathogenesis. “Intestinal Dysbiosis Associated with Systemic Lupus Erythematosus” (741 citations) systematically demonstrated how gut dysbiosis contributes to SLE through molecular mimicry, impaired gut barrier function, and immune modulation.

**Figure 2.**
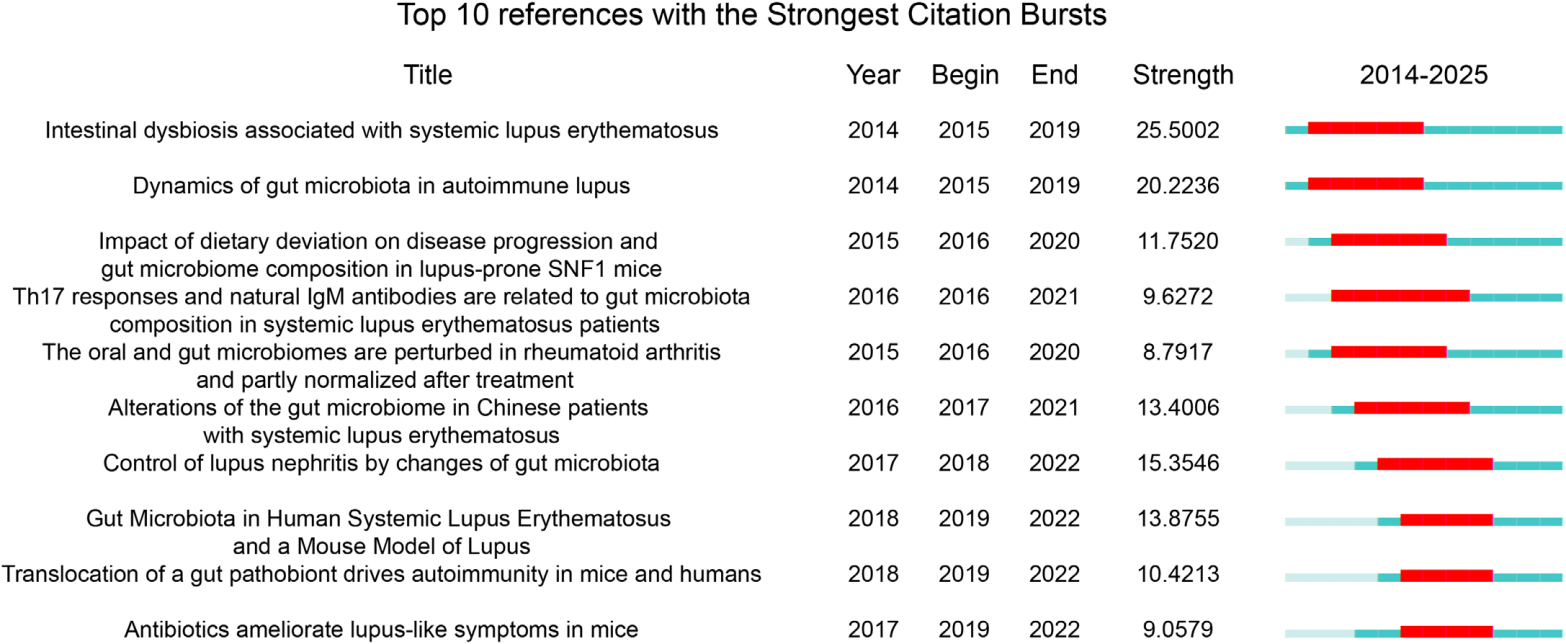
Top cited publications and citation burst analysis in systemic lupus erythematosus–gut microbiota research.

Citation burst analysis identified publications with strong temporal influence (Figure 2). “Intestinal Dysbiosis Associated with Systemic Lupus Erythematosus” exhibited the strongest burst (citation burst = 25.5002) between 2015 and 2019, followed by “Dynamics of Gut Microbiota in Autoimmune Lupus” (burst = 20.2236, 2015-2019) and “Control of Lupus Nephritis by Changes of Gut Microbiota” (burst = 15.3546, 2018-2022). These studies collectively mark key turning points in the evolution of this research field. Detailed citation burst values and corresponding time periods are summarized in Supplementary Table 2.

### Keyword Analysis and Thematic Evolution

**Figure 3.**
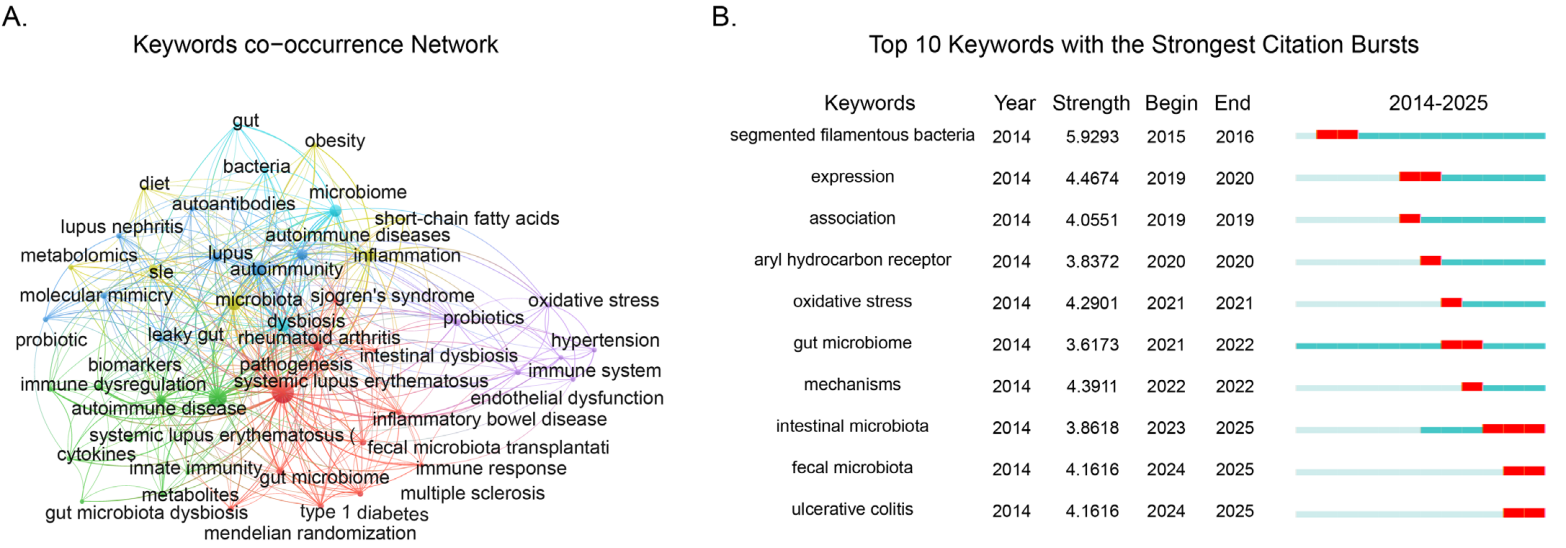
Keyword analysis in systemic lupus erythematosus–gut microbiota research. (A) Keyword co-occurrence network. (B) Keywords with the strongest citation bursts (2014-2025).

Keyword co-occurrence analysis identified 47 high-frequency keywords (occurrence > 5), visualized in Figure 3A. The most frequently occurring terms were SLE (n = 293), gut microbiota (n = 227), and intestine flora (n = 145). Co-occurrence analysis revealed the strongest association between gut microbiota and SLE (154 co-occurrences), followed by SLE and intestine flora (143 co-occurrences).

Keyword burst analysis (Figure 3B) revealed emerging research hotspots over time. Segmented filamentous bacteria showed the strongest burst (burst = 5.9293, 2015-2016), followed by expression (burst = 4.4674, 2019-2020) and mechanisms (burst = 4.3911, 2022-2022). Temporal keyword analysis further indicated a clear thematic evolution: early studies (2014-2016) focused on exploratory microbial profiling, especially segmented filamentous bacteria; subsequent research (2017-2019) emphasized bacterial metabolites, mainly focusing on short-chain fatty acids; and recent studies (2020 onward) increasingly concentrated on dysbiosis-driven immune regulation and inflammation. This progression reflects a systematic shift from descriptive analyses toward mechanistic and translational research, such as fecal microbiota transplantation, drug therapy, and etiology. The keyword co-occurrence matrix, detailed burst statistics, and annual keyword distributions are provided in Supplementary Tables 3-5.

## Discussion

### Research Trends and Growth Dynamics

This bibliometric analysis demonstrates a clear stage-wise evolution of research on the association between SLE and the gut microbiota. From 2014 to 2018, the field remained in an exploratory phase, characterized by relatively low annual publication output and a primary focus on descriptive microbial profiling.^^28^^ Early studies mainly sought to identify compositional differences in gut microbiota between patients with SLE and healthy controls, laying the conceptual foundation for subsequent investigations.

A marked acceleration in publication output was observed after 2019, coinciding with an increasing number of experimental studies exploring the potential role of gut dysbiosis in lupus pathogenesis. In particular, animal studies demonstrating that modulation of gut microbiota could influence autoantibody production and disease severity provided a critical turning point, stimulating widespread interest in microbiota–immune system interactions.^^29^^ Together, these findings suggest a transition from hypothesis-driven exploration to more mechanism-oriented and translational research.

### Geographic Distribution and Collaborative Patterns

The analysis identified China and the United States as the 2 leading contributors to SLE–gut microbiota research, collectively accounting for more than half of the total publications. Despite their comparable productivity, notable differences were observed in research focus. Institutions in the United States have predominantly emphasized mechanistic investigations, exploring links between gut microbiota, intestinal permeability, inflammatory mediators, and systemic immune activation. In contrast, Chinese research groups have focused more extensively on clinical association studies, particularly characterizing microbiota composition and diversity in patients with SLE.^^30^^

International collaboration analysis revealed that China–United States cooperation represents the most prominent collaborative axis within this field, suggesting a potentially complementary relationship between mechanistic and clinical research approaches. Although European countries contributed a smaller proportion of publications overall, their relative strength in microbiome-related metabolomic studies underscores the potential value of broader multinational collaborations. Enhanced cross-regional cooperation may facilitate the integration of mechanistic insights with population-based clinical evidence.

### Evolution of the Knowledge Structure and Emerging Research Fronts

Keyword evolution and citation burst analyses revealed 3 major thematic shifts in the intellectual structure of this field. During the initial phase, research was largely driven by immuno-inflammatory hypotheses linking SLE and gut microbiota, with emphasis on microbial composition and diversity. Subsequently, studies increasingly focused on mechanistic pathways, including molecular mimicry, epithelial barrier dysfunction, and immune modulation. More recently, research attention has shifted toward translational themes, such as dysbiosis-driven immune regulation and therapeutic modulation of the gut microbiota.

Notably, emerging keywords related to specific bacterial taxa and autoantibody production highlight a growing interest in identifying microbial drivers of immune dysregulation in SLE. This evolution reflects a broader shift in research focus from descriptive associations toward mechanism-oriented and intervention-related investigations, aligning with current priorities in autoimmune disease research.

### Implications for Pathogenesis and Clinical Translation

The progressive refinement of research themes observed in this bibliometric analysis indicates growing scientific interest in the potential involvement of gut microbiota in SLE. Existing experimental evidence and clinical studies have explored associations, suggesting that specific microbial taxa and their metabolites can influence pro-inflammatory and regulatory immune pathways, alter autoantibody production, and modulate disease activity.^31^,^32^^-^^^34^^ In addition, recent reviews have highlighted the broader role of the microbiome in rheumatic diseases, further supporting the relevance of microbiota–immune interactions in autoimmune conditions.^^35^^ However, it should be emphasized that bibliometric analysis itself reflects research dynamics rather than providing direct mechanistic or causal evidence.

The increasing prominence of keywords related to probiotics, dietary interventions, and microbiota modulation highlights expanding translational exploration within the field. However, translating these approaches into clinical practice remains challenging. Variability in microbiota composition across populations, differences in disease phenotype, and the lack of standardized intervention protocols currently limit clinical applicability. Future integrative and longitudinal investigations may further clarify the therapeutic potential of microbiota-based strategies in SLE.

### Limitations and Future Perspectives

Several limitations of this study should be acknowledged. First, the analysis was restricted to publications indexed in the Web of Science Core Collection and the Scopus database, and limited to English-language articles, potentially underrepresenting research output from non-English-speaking regions. Therefore, the global research output and collaboration patterns reported in this study should be interpreted with this limitation in mind. Second, only original research articles and reviews were included, excluding conference proceedings, preprints, and patent data, which may contain emerging or translational insights. Finally, the bibliometric approach emphasizes quantitative patterns and does not provide in-depth qualitative evaluation of individual studies.

Despite these limitations, this study offers a comprehensive overview of the global research landscape on SLE and gut microbiota. Future research should prioritize large-scale, population-based studies and integrative multidisciplinary approaches to better elucidate the causal mechanisms linking gut dysbiosis and autoimmune responses. Strengthening international collaboration and bridging basic research with clinical investigation may ultimately facilitate the development of microbiota-based therapeutic strategies for patients with SLE.

## Conclusion

This bibliometric analysis provides an overview of the global research landscape concerning studies on SLE and the gut microbiota from 2014 to 2025. The findings suggest a growing body of literature and an apparent shift from descriptive microbial profiling toward mechanistic and translational research. China and the United States emerged as major contributors, with complementary research focuses that underscore the value of international collaboration. Overall, this study offers a structured overview of publication trends and thematic evolution and may help inform future research directions in this field.

### Data Availability Statement:

The datasets used and/or analyzed during the current study are available from the corresponding author on reasonable request.

### Artificial Intelligence Usage Statement:

During the preparation of this manuscript, the authors used ChatGPT (OpenAI) to assist with language editing and improving the clarity and readability of the text. However, all content of the study has been reviewed and edited by the authors following the use of Al/LLM tools. The authors take full responsibility for the scientific content, interpretation, and conclusions presented in the article.

### Ethics Committee Approval:

N/A.

### Informed Consent:

N/A.

### Peer-review:

Externally peer-reviewed.

### Author Contributions:

Concept – J.W., J.Y.; Design – J.W.; Data Collection and/or Processing – J.Y.; Analysis and/or Interpretation – J.W., J.Y.; Literature Search – J.Y.; Writing – J.Y., J.W.; Critical Review – J.W.

### Declaration of Interests:

The authors have no conflicts of interest to declare.

### Funding:

The authors declare that this study received no financial support.

## Supplementary Materials

Supplementary Material

## Figures and Tables

**Table 1.** International Collaboration Networks Among Leading Countries in Systemic Lupus Erythematosus–Gut Microbiota Research

**Nation**	**Number of Articles Published**	**Field-Normalized Contribution**	**Partnership**	**Number of Co-Operation**
China	172	1.0927	China, USA	21
USA	103	1.2268	USA, Germany	9
Italy	37	0.9451	China, United Kingdom	5
Spain	30	0.6996	Spain, Italy	5
Japan	12	0.7348	USA, Italy	4
Brazil	12	0.6847	USA, United Kingdom	4
Iran	12	0.9640	China, Australia	4
Korea	11	0.9283	Israel, Russia	3
United Kingdom	11	0.4537	USA, Brazil	3
India	7	0.5647	USA, Egypt	3

**Table 2.** Top 10 Journals Publishing Studies on Systemic Lupus Erythematosus and Gut Microbiota

**Journal**	**Country**	**Articles**	****Impact Factor** (2024-2025)**	**Journal Citation Reports - Category**	**Number of Citations**	**Average Number of Citations**
*Frontiers in Immunology*	Switzerland	67	5.9	Q1	1368	20.4179
*International Journal of Molecular Sciences*	USA	19	4.9	Q1	346	18.2105
*Journal of Autoimmunity*	United Kingdom	16	7.0	Q1	556	34.7500
*Autoimmunity Reviews*	USA	13	8.3	Q1	369	28.3846
*Frontiers in Cellular and Infection Microbiology*	Switzerland	13	4.8	Q1	139	10.6923
*Frontiers in Microbiology*	Switzerland	13	4.5	Q1	313	24.0769
*Nutrients*	Switzerland	9	5.0	Q1	347	38.5555
*Scientific* * Reports*	United Kingdom	7	3.9	Q1	602	86.5714
*Microorganisms*	Switzerland	6	4.2	Q1	88	14.6667
*Gut Microbes*	USA	6	11.0	Q1	280	46.6667

**Table 3.** Top 10 Most Cited References in Systemic Lupus Erythematosus–Gut Microbiota Research

**Title**	**Journal**	**Total Citations**
The Bidirectional Relationship of Depression and Inflammation: Double Trouble	*Neuron*	1598
Does the Epithelial Barrier Hypothesis Explain the Increase in Allergy, Autoimmunity, and Other Chronic Conditions?	*N* *ature Reviews Immunology*	851
Intestinal Dysbiosis Associated with Systemic Lupus Erythematosus	*mBio*	741
Leaky Gut as a Danger Signal for Autoimmune Diseases	*Frontiers in Immunology*	488
The Microbiome in Autoimmune Diseases	*Clinical and Experimental Immunology*	486
The Gut Microbiota in Immune-Mediated Inflammatory Diseases	*Frontiers in Microbiology*	463
Lupus Nephritis Is Linked to Disease-Activity Associated Expansions and Immunity to a Gut Commensal	*Annals of the Rheumatic Diseases*	450
Gut Microbiota-Derived Metabolites in the Regulation of Host Immune Responses and Immune-Related Inflammatory Diseases	*Cellular and Molecular Immunology*	443
Control of Lupus Nephritis by Changes of Gut Microbiota	*Microbiome*	440
The Oral Microbiota Is Modified by Systemic Diseases	*Journal of Dental Research*	405
