# Supplementary material for: Research Trends and Emerging Themes in Gut Microbiota–Systemic Lupus Erythematosus Research: A Bibliometric Analysis (2014-2025)
Source: Arch Rheumatol. 2026 Jun 3;41(3):249–57. doi: 10.5152/ArchRheumatol.2026.25284 (PMC13401116; doi:10.5152/ArchRheumatol.2026.25284)
Supplement: Supplementary Material [file supplementary_material.pdf]

Supplementary Table 1. Top 20 most productive institutions involved in systemic lupus erythematosus–gut microbiota research

Institutions	Country	Articles
Zhejiang Chinese Medical University	China	10
Medical University Of South Carolina	USA	10
Virginia Polytechnic Institute And State University	USA	8
Central South University	China	6
Chulalongkorn University	Thailand	6
Anhui Medical University	China	6
Southern Medical University - China	China	6
Catholic University Of Korea	Korea	5
Csic - Instituto De Productos Lacteos De Asturias (Ipla)	Spain	5
Edward Via College Of Osteopathic Medicine (Vcom)	USA	5
University Of Granada	Spain	5
Beijing Hospital	CHINA	4
New York University	USA	4
Peking Union Medical College	China	4
Peking Union Medical College Hospital	CHINA	4
Shanxi Medical University	CHINA	4
Universidade De Sao Paulo	Brazil	4
University Of Oviedo	Spain	4
Baylor College Of Medicine	USA	3
Beijing University Of Chinese Medicine	China	3

Supplementary Table 2. Publications with the strongest citation bursts identified by CiteSpace

Begin	End	Strength	Year	Citation information
2015	2019	25.5002	2014	Hevia A, 2014, MBIO, V5, P0, DOI 10.1128/mBio.01548-14
2015	2019	20.2236	2014	Zhang HS, 2014, APPL ENVIRON MICROB, V80, P7551, DOI 10.1128/AEM.02676-14
2016	2020	11.752	2015	Johnson BM, 2015, CLIN EXP IMMUNOL, V181, P323, DOI 10.1111/cei.12609
2016	2021	9.6272	2016	López P, 2016, SCI REP-UK, V6, P0, DOI 10.1038/srep24072
2016	2020	8.7917	2015	Zhang X, 2015, NAT MED, V21, P895, DOI 10.1038/nm.3914
2017	2021	13.4006	2016	He ZX, 2016, GUT PATHOG, V8, P0, DOI 10.1186/s13099-016-0146-9
2018	2022	15.3546	2017	Mu QH, 2017, MICROBIOME, V5, P0, DOI 10.1186/s40168-017-0300-8
2019	2022	13.8755	2018	Luo XM, 2018, APPL ENVIRON MICROB, V84, P0, DOI 10.1128/AEM.02288-17
2019	2022	10.4213	2018	Vieira SM, 2018, SCIENCE, V359, P1156, DOI 10.1126/science.aar7201
2019	2022	9.0579	2017	Mu QH, 2017, SCI REP-UK, V7, P0, DOI 10.1038/s41598-017-14223-0

Supplementary Table 3. High-frequency keyword co-occurrence matrix

Keywords	Articles	co-occurrence pair of keywords	Times
systemic lupus erythematosus	293	gut microbiota; systemic lupus erythematosus	154
gut microbiota	227	systemic lupus erythematosus; intestine flora	143
intestine flora	145	systemic lupus erythematosus; human	139
human	139	gut microbiota; dysbiosis	137
dysbiosis	128	T-cells; pathogenesis	117
autoimmunity	118	systemic lupus erythematosus; autoimmunity	113
humans	113	systemic lupus erythematosus; gastrointestinal microbiome	110
gastrointestinal microbiome	111	autoimmunity; systemic lupus erythematosus	109
inflammation	105	systemic lupus erythematosus; inflammation	108
nonhuman	103	gut microbiota; inflammation	107

Supplementary Table 4. Top keywords with citation bursts and corresponding time periods

Begin	End	Strength	Year	Keywords
2015	2016	5.9293	2014	segmented filamentous bacteria
2019	2020	4.4674	2014	expression
2019	2019	4.0551	2014	association
2020	2020	3.8372	2014	aryl hydrocarbon receptor
2021	2021	4.2901	2014	oxidative stress
2021	2022	3.6173	2014	gut microbiome
2022	2022	4.3911	2014	mechanisms
2023	2025	3.8618	2014	intestinal microbiota
2024	2025	4.1616	2014	fecal microbiota
2024	2025	4.1616	2014	ulcerative colitis

Supplementary Table 5. Annual distribution of high-frequency keywords (2014-2025)

Term	Frequency	Year (Q1)	Year (Median)	Year (Q3)
Segmented filamentous bacteria	11	2016	2016	2020
innate lymphoid-cells	5	2016	2016	2020
commensal microbiota	7	2016	2017	2021
murine lupus	5	2015	2017	2020
priority journal	23	2018	2018	2019
crohns-disease	9	2017	2018	2023
disease association	8	2017	2018	2020
classification criteria	7	2018	2019	2022
periodontitis	7	2019	2019	2020
short-chain fatty acids	7	2018	2019	2024
systemic-lupus-erythematosus	88	2017	2020	2023
gastrointestinal tract	20	2018	2020	2022
dysbiosis	16	2019	2020	2024
autoimmunity	118	2019	2021	2023
microbiota	96	2019	2021	2023
systemic lupus erythematosus	291	2020	2022	2024
intestine flora	145	2020	2022	2024
gut microbiota	227	2020	2023	2024
fecal microbiota transplantation	44	2020	2023	2024
microbial community	24	2022	2024	2024
systematic review	22	2022	2024	2025
animal model	20	2022	2024	2025
therapy	16	2024	2025	2025
drug therapy	8	2024	2025	2025
etiology	7	2024	2025	2025
